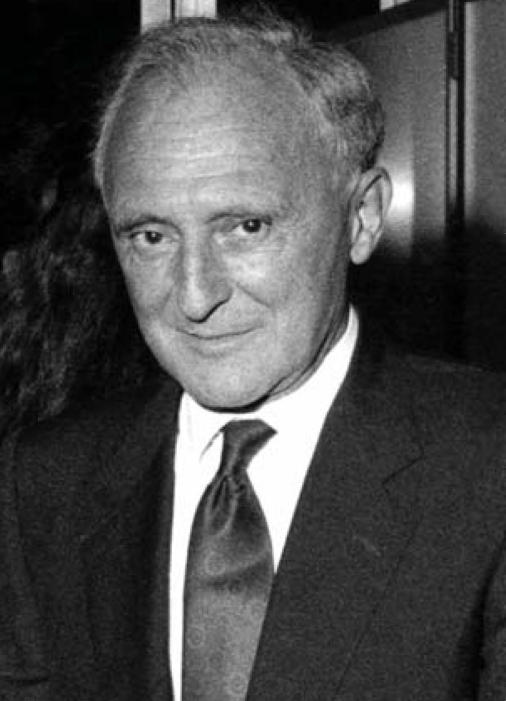# Lorenzo Tomatis 1929–2007

**DOI:** 10.1289/ehp.116-a16

**Published:** 2008-01

**Authors:** James Huff, Ronald Melnick

**Affiliations:** National Institute of Environmental Health Sciences, National Institutes of Health Department of Health and Human Services, Research Triangle Park, North Carolina, E-mail: huff1@niehs.nih.gov

On Friday, 21 September 2007, in Lyon, France, we lost a great human being, a staunch advocate for public health, a thorough and delving scientist, and a humanitarian par excellence. Lorenzo Tomatis, MD, above all, was a learned teacher and creative innovator. His accomplishments are legion, and his far-reaching impact on human health, including the well-being of future generations, will be impossible to replace. Tomatis was clearly a true pioneer and admired leader in primary disease prevention. He stands tall among other giants and trailblazers of environmental health science and public health advocacy including Cesare Maltoni, Norton Nelson, David Rall, and Irving Selikoff. At the same time, Tomatis was respected, admired, and loved by his colleagues and fellow public health advocates as a man whose warmth, humor, strength, and sweetness were as compelling as his command of science.

During his tenure at the International Agency for Research on Cancer (IARC), Tomatis was instrumental in creating what has come to be recognized and known as the monument of primary prevention of cancer: the *IARC Monographs on the Evaluation of Carcinogenic Risk to Humans* (also known as the “orange books”). Since 1972, when the first monograph was published, this series of nearly 100 volumes has provided objective evaluations of carcinogenicity on agents, mixtures, and exposure circumstances. Not only did Tomatis design and develop the *IARC Monographs*, he was a principal leader in the scientific community in recognizing the essential value of transplacental and transgenerational carcinogenesis and developing methodologies to determine the influence of early-life exposures. Then and over time, these innovations spurred further primary prevention strategies of action and additional experimental derivatives. As a champion of modern carcinogenesis bioassays, Tomatis led the difficult course of defining the applicability and utility of these experimental animal findings for preventing cancers in humans, especially in workers and children.

Tomatis was born in Sassoferrato, Italy, and began his professional career upon graduating from the University of Turin with a degree in medicine in 1953, in hygiene and preventive medicine in 1955, and in occupational health in 1957. Tomatis recognized early on that, although he enjoyed serving the medical needs of individuals, his passion for fostering public health, merged with scientific undergirding, led him to new and different challenges. In 1959, newly arrived at the University of Chicago, he began studying occupational disease and became immersed in cancer research, particularly cancer causation. Almost immediately he recognized the obvious value and significance of preventing disease, particularly occupational cancers, rather than relying on the then-current direction of cancer diagnoses and treatment. His lifework was ultimately dedicated to this vital mission.

In 1967, Tomatis moved to Lyon, France, as one of the first leadership employees of the newly established IARC. Upon arriving, he formed the Unit of Chemical Carcinogenesis. There he envisioned and began to effectuate more than a monolithic cancer research program—he insisted on a solid effort on primary prevention of cancer. His view on prevention came to fruition in 1972 with the publication of the first volume of the *IARC Monographs*. This public health breakthrough had instantaneous and, as we now know, enduring impact throughout the world. In fact, IARC quickly became known predominantly for the *IARC Monographs*, its most visible and useful product. This international recognition of the value and authority of the *IARC Monographs* was bolstered by the impartial and scientific process initiated by Tomatis for evaluating chemical carcinogens. Tomatis and IARC staff brought together working groups of independent scientists with vast knowledge of chemical carcinogenesis to evaluate the available information used to prepare the objective *IARC Monographs*.

In January 1982, in recognition of his outstanding career and contributions in understanding cancer causation and prevention, Tomatis was elected by the World Health Organization (WHO) member nations as the second director of IARC. He was reelected and served as director for a full 12 years, until his retirement in December 1993. During his tenure at IARC, Tomatis and the international IARC staff developed a mutual respect and appreciation for each other and were unified in promoting the agency’s mission of improving public health through primary disease prevention. Upon retirement from IARC/WHO, he served as scientific director of the Institute of Child Health “Burlo Garofolo” in Trieste, Italy, from 1996 to 1999.

Shortly thereafter, Tomatis joined the National Institute of Environmental Health Sciences (NIEHS) in North Carolina to focus on issues that he had little time to pursue while serving as the director of IARC. Under the International Scholar Program, Tomatis worked at the NIEHS for six summers, sharing his professional experience and writing important papers on environmental public health and prevention. During that time, he was also the chairman of the Scientific Committee of the International Society of Doctors for the Environment in Arezzo, Italy.

Tomatis was born in Italy, worked in the United States, and spent most of his professional career in France; he was a person of immense global scientific and public health impact. Able to speak four languages—Italian, French, English, and German—Tomatis traveled the world to spread his undiluted message of primary prevention of diseases, especially cancer. He was as prolific as he was dedicated to protecting the environment, including workplaces and public health, and he was an early and long-standing champion of environmental and social justice. Much of his science is captured in 10 books and more than 350 scientific papers. Being a renaissance man, Tomatis also wrote books for the public based on his medical, scientific, and humanitarian experiences: *The Unlimited Research* 1974, *Seen from the Inside* 1981, *Natural History of the Researcher* 1985, *The Laboratory* 1993, *The Reelection* 1996, and *The Refugee* 2005.

His gifts to humanity are well recognized in saved lives and a cadre of scientists influenced by his magnanimity, honesty, sense of ethics, and moral character. Most will remember Tomatis for his brilliance in science and public health thought, but he will more likely be venerated for his generosity and compassion. His heroic scientific determination and his adventurous spirit and love of people inspired Tomatis to live life to its fullest. He is survived by his wife, Delia, in Trieste, and their son, Paolo, who lives and works in Rome.

His gifts to humanity are well recognized in saved lives and a cadre of scientists influenced by his magnanimity, honesty, sense of ethics, and moral character.

## Figures and Tables

**Figure:**